# Purification, Identification and Characterization of Antioxidant Peptides from Corn Silk Tryptic Hydrolysate: An Integrated In Vitro-In Silico Approach

**DOI:** 10.3390/antiox10111822

**Published:** 2021-11-17

**Authors:** Joe-Hui Ong, Jiun-An Koh, Hui Cao, Sheri-Ann Tan, Fazilah Abd Manan, Fai-Chu Wong, Tsun-Thai Chai

**Affiliations:** 1 Department of Chemical Science, Faculty of Science, Universiti Tunku Abdul Rahman, Kampar 31900, Malaysia; candy3997@1utar.my (J.-H.O.); jiunan30@1utar.my (J.-A.K.); wongfc@utar.edu.my (F.-C.W.); 2 College of Food Science and Technology, Guangdong Ocean University, Zhanjiang 524088, China; hui_cao0830@yahoo.com; 3 Department of Bioscience, Faculty of Applied Sciences, Tunku Abdul Rahman University College, Setapak, Kuala Lumpur 53300, Malaysia; tansw@tarc.edu.my; 4 Department of Biosciences, Faculty of Science, Universiti Teknologi Malaysia, Skudai, Johor Bahru 81310, Malaysia; m-fazilah@utm.my; 5 Center for Agriculture and Food Research, Universiti Tunku Abdul Rahman, Kampar 31900, Malaysia

**Keywords:** antioxidant, in silico, in vitro, mechanism, molecular docking, peptide, purification, *Stigma maydis*

## Abstract

Corn silk (CS) is an agro-by-product from corn cultivation. It is used in folk medicines in some countries, besides being commercialized as health-promoting supplements and beverages. Unlike CS-derived natural products, their bioactive peptides, particularly antioxidant peptides, are understudied. This study aimed to purify, identify and characterize antioxidant peptides from trypsin-hydrolyzed CS proteins. Purification was accomplished by membrane ultrafiltration, gel filtration chromatography, and strong-cation-exchange solid-phase extraction, guided by 2,2′-azino-bis(3-ethylbenzothiazoline-6-sulfonic acid) diammonium salt radical cation (ABTS^•+^) scavenging, hydrogen peroxide scavenging, and lipid peroxidation inhibition assays. De novo sequencing identified 29 peptides (6–14 residues; 633–1518 Da). The peptides consisted of 33–86% hydrophobic and 10–67% basic residues. Molecular docking found MCFHHHFHK, VHFNKGKKR, and PVVWAAKR having the strongest affinity (−4.7 to −4.8 kcal/mol) to ABTS^•+^, via hydrogen bonds and hydrophobic interactions. Potential cellular mechanisms of the peptides were supported by their interactions with modulators of intracellular oxidant status: Kelch-like ECH-associated protein 1, myeloperoxidase, and xanthine oxidase. NDGPSR (Asn-Asp-Gly-Pro-Ser-Arg), the most promising peptide, showed stable binding to all three cellular targets, besides exhibiting low toxicity, low allergenicity, and cell-penetrating potential. Overall, CS peptides have potential application as natural antioxidant additives and functional food ingredients.

## 1. Introduction

Corn silk (*Stigma maydis*) (CS) is the thread-like style at the top of an ear of corn. Although CS is discarded as an agricultural by-product worldwide, the value of CS as an herbal remedy has been recognized in the traditional medicines of some countries [1]. At present, CS health supplements and CS-based tea are also available to consumers. Phytochemically, CS is rich in flavonoids, which are responsible for some bioactivities of CS, such as antioxidant, anti-fatigue, and anti-hyperlipidemic [1]. In contrast to natural product exploration, the identification and characterization of CS-derived bioactive peptides are limited. CS consists of 17.6% of crude proteins by dry weight [1], hence it may be a potential source of bioactive peptides. To date, only antihypertensive [2] and anti-inflammatory [3] peptides were identified from CS, but there is no report of CS-derived antioxidant peptides. In our previous study, we found CS tryptic hydrolysate to be a more potent scavenger of hydrogen peroxide (H_2_O_2_) and superoxide than glutathione (GSH) and carnosine, two well-established peptidic antioxidants. Moreover, CS tryptic hydrolysate was more effective than the two aforementioned antioxidants in protecting human red blood cells from oxidative injury [4]. Nevertheless, the identity of the antioxidant peptides in the hydrolysate has not been unraveled.

Antioxidant peptides derived from food and agricultural waste/by-products are recognized for their potential applications as food additives, functional food, health-promoting supplements, and lead compounds for drug discovery. In the food industry, such peptidic antioxidants of natural origin could be substitutes for synthetic antioxidants, which have raised concerns about health risks. Besides attenuating food oxidation, antioxidant peptides could also serve as curative or preventive agents of reactive oxygen species (ROS)-mediated diseases, supporting the application of such peptides in functional food and health supplement formulation [5]. Antioxidant peptides could alleviate cellular oxidative damage by scavenging free radicals and/or by regulating the cellular production of antioxidants or oxidants. The Keap1/Nrf2 pathway is a major pathway that modulates cellular antioxidant responses, which can be activated by antioxidant peptides [6]. Under oxidative stress, nuclear factor erythroid 2-related factor 2 (Nrf2) bound to Kelch-like ECH-associated protein 1 (Keap1) will detach and migrate to the nucleus, where it activates the expression of antioxidant genes [7]. By contrast, myeloperoxidase (MPO) and xanthine oxidase (XO) are associated with cellular ROS production. Antioxidant peptides isolated from fish skin gelatin hydrolysate alleviated oxidative injury in mice by inhibiting MPO, besides activating the Nrf2 to upregulate the expression of antioxidant enzymes [8]. Antioxidant peptides derived from mackerel meat, egg white, and tuna backbone protein were also demonstrated as potent XO inhibitors [9].

In this study, we adopted an integrated in vitro-in silico approach to purify, identify, and characterize antioxidant peptides from a CS tryptic hydrolysate. Purification using membrane ultrafiltration (UF), gel filtration chromatography (GFC), and strong-cation-exchange solid-phase extraction (SCX-SPE) were guided by antioxidant assays including 2,2′-azino-bis(3-ethylbenzothiazoline-6-sulfonic acid) diammonium salt radical cation (ABTS^•+^) scavenging, H_2_O_2_ scavenging, and lipid peroxidation inhibition assays. The peptides identified by de novo peptide sequencing were subjected to in silico analyses. Physicochemical properties of the peptides were analyzed to clarify the relationship between amino acid composition and the antioxidant potential of the CS hydrolysate-derived peptides. Toxicity, allergenicity, and cell-penetrating potential of the peptides were predicted. Moreover, molecular docking simulation was performed to investigate the antioxidant mechanisms of the peptides in the context of their intermolecular interactions with ABTS^•+^ and with the modulators of cellular oxidant status Keap1, MPO, and XO.

## 2. Materials and Methods

### 2.1. Materials and Reagents

CS was provided by Mr. Khuan Thean Ang, the owner of a local corn plantation located at Pinang Tunggal, Pulau Pinang, Malaysia. *O*-phthalaldehyde (OPA) and trypsin (from hog pancreas, 1539 U/mg) were purchased from Nacalai Tesque Inc. (Kyoto, Japan). GSH was purchased from Sigma-Aldrich (Saint Louis, MO, USA). Lecithin (egg) and trichloroacetic acid were purchased from Fisher Scientific (Waltham, MA, USA). Copper (II) acetate monohydrate and H_2_O_2_ were purchased from R & M Chemicals (London, UK). Formic acid, acetonitrile, 2,2′-azino-bis(3-ethylbenzthiazoline-6-sulfonic acid) (ABTS) diammonium salt, thiobarbituric acid, and UF centrifugal filter unit with 3 kDa molecular weight cut-off (MWCO) were purchased from Merck (Darmstadt, Germany). Sephadex G-25 resins were purchased from GE Healthcare (Danderyd, Sweden). Solid-phase extraction (SPE) cartridges STRATA SCX (sorbent mass: 1000 mg; volume: 6 mL) and STRATA C18-E (sorbent mass: 500 mg; volume: 3 mL) were purchased from Phenomenex Inc. (Torrance, CA, USA). All the reagents and solvents were of analytical grade.

### 2.2. Preparation of CS Protein Isolate and Hydrolysate

CS protein isolate and 1-h trypsin hydrolysate (T1H) were prepared as previously described [4]. Peptide contents were determined by using the OPA assay [10].

### 2.3. Purification of Antioxidant Peptides from T1H

T1H was purified by using UF, GFC, and SCX-SPE as previously reported [11], with modifications. T1H, dissolved in deionized water, was fractionated by using UF centrifugal filter units (MWCO 3 kDa). Both the retentate and permeate fractions, designated as >3 kDa UF and <3 kDa UF, were collected and lyophilized. The <3 kDa UF fraction was further separated on a Sephadex G-25 chromatographic column (1.6 × 70.0 cm, Sigma-Aldrich, Saint Louis, MO, USA), eluted with deionized water (flow rate 0.6 mL/min). The resultant fractions were monitored spectrophotometrically at 280 nm and subsequently combined into three pooled fractions designated as GF-I, GF-II, and GF-III. The lyophilized GF-III fraction was next purified by using a STRATA SCX cartridge (Phenomenex Inc., Torrance, CA, USA) as described previously [11]. 

Protein contents of the UF fractions were determined by using the Bradford reagent (Bio Basic Inc., Markham, Canada) [12]. Quantification of peptides in fractions collected during the aforementioned purification steps were accomplished with the OPA assay [10]. Antioxidant activities were evaluated by using ABTS^•+^ and H_2_O_2_ scavenging assays, as well as lipid peroxidation inhibition assay as described below.

### 2.4. Identification of Purified Peptides

Following the SCX-SPE purification step, the resultant desalted 0, 20, and 200 mM KCl fractions were selected for de novo peptide sequencing based on a liquid chromatography-tandem mass spectrometry (LC-MS/MS) system (Thermo Fisher Scientific, Waltham, MA, USA). The analysis was performed at the Proteomics Laboratory, Malaysia Genome and Vaccine Institute, National Institutes of Biotechnology Malaysia. The Ultimate 3000 RSLC nano-high-performance liquid chromatography system (Thermo Fisher Scientific, Waltham, MA, USA), coupled to an Orbitrap Fusion mass spectrometer (Thermo Fisher Scientific, Waltham, MA, USA), was used to analyze the samples. Peptides were sequenced by using the DeNovoGUI Software (Version 1.16.2, Max Planck Institute, Munich, Germany), based on the MS/MS spectral data collected.

### 2.5. Determination of Antioxidant Activities

The ABTS^•+^ scavenging activity of peptide samples was determined by using a decolorization assay, as previously reported [11]. The H_2_O_2_ scavenging activity was evaluated as previously outlined [13], except that 600 μL H_2_O_2_ (40 mM) was included in the reaction mixture. The protective effect against lipid peroxidation was estimated by using a lecithin liposome model [4]. Briefly, a mixture containing the test sample, lecithin, and copper (II) acetate monohydrate was incubated in darkness at 37 °C for 24 and 48 h, followed by monitoring of absorbance at 532 and 600 nm. Thiobarbituric acid reactive species (TBARS) levels were calculated and expressed as μM malondialdehyde (MDA) equivalents. GSH was used as a reference antioxidant in the three antioxidant assays.

### 2.6. In Silico Analysis

#### 2.6.1. Modelling of Peptide Structures

The three-dimensional (3D) conformations of peptides identified by the LC-MS/MS experiment were modeled with PEP-FOLD 3.5 (https://bioserv.rpbs.univ-paris-diderot.fr/services/PEP-FOLD3) (access date: 12 June 2021). PEP-FOLD 3.5 is an online tool for the de novo prediction of peptide structures from amino acid sequences [14,15,16]. The tasks were set to allow 200 simulation runs and the conformation models were evaluated based on sOPEP energy scores. The best-scored model for each peptide was saved in the Protein Data Bank (PDB) format and used in molecular docking analysis.

#### 2.6.2. Prediction of Antioxidant Peptides and Docking to ABTS^•+^

Peptide sequences identified by the LC-MS/MS experiment were submitted to the AnOxPePred web server (https://services.healthtech.dtu.dk/service.php?AnOxPePred-1.0) [17] (accessed on 18 June 2021) to predict free radical scavenging activities. Based on their scores, selected peptides were used in molecular docking analysis. 

For docking, the 3D conformer of ABTS (PubChem CID: 5360881) was downloaded from PubChem (https://pubchem.ncbi.nlm.nih.gov) [18] (access date: 15 June 2021) in the Structure Data File (SDF) format and converted to the PDB format by using the BIOVIA Discovery Studio Visualizer (BIOVIA, Dassault Systèmes, BIOVIA Discovery Studio Visualizer, Version 20.1.0.192, San Diego, CA, USA: Dassault Systèmes, 2020). The PDB coordinates of ABTS were processed for energy minimization by using the GlycoBioChem PRODRG2 server (http://davapc1.bioch.dundee.ac.uk/cgi-bin/prodrg) [19] (access date: 15 June 2021). The resultant PDB file was prepared as a ligand in the PDBQT format with AutoDockTools 1.5.6 (The Scripps Research Institute, La Jolla, CA, USA) [20,21]. Peptides were prepared for docking by adding Kollman charges and polar hydrogens by using AutoDockTools 1.5.6, saved in the PDBQT format. Docking simulation was performed by using default settings on Webina 1.0.2 (https://durrantlab.pitt.edu/webina) [22] (access date: 16 June to 26 October 2021). Coordinates of docking-box center and box size are provided in Table S1. Through preliminary optimization trials, the box settings were chosen to center on the peptide structure being docked, ensuring the box was large enough to enclose the whole peptide. The two-dimensional (2D) diagrams of peptide-ABTS^•+^ interactions were visualized by using LigPlot+ v.2.2 (EMBL-EBI, Cambridge, UK) [23,24].

#### 2.6.3. Docking-Based Screening of Potential Inhibitors of Keap1, MPO, and XO

The X-ray crystal structures of murine Keap1 in complex with inhibitor RA839 (PDB code: 5CGJ) [7], human MPO bound to inhibitor 7-benzyl-1*H*-[1,2,3]triazolo [4,5-b]pyridin-5-amine (PDB code: 6WYD) [25], and bovine XO complexed with quercetin (PDB code: 3NVY) [26] were downloaded from the RCSB Protein Data Bank (https://www.rcsb.org) [27,28] (access date: 19 June 2021). Proteins and the bound ligands were separated by using BIOVIA Discovery Studio Visualizer (Dassault Systèmes, San Diego, CA, USA). The three proteins were prepared as receptors, whereas the CS peptides to be docked were prepared as ligands by using AutoDockTools 1.5.6, all saved in the PDBQT format as previously described [29]. The docking of CS peptides to these three protein targets was performed by using Webina 1.0.2 (University of Pittsburgh, Pittsburgh, PA, USA). To validate our docking protocol, redocking of the crystallographic ligands to the respective proteins were performed and the root mean square deviation (RMSD) were obtained. Coordinates of the docking-box center and box size, as well as the RMSD values obtained, are provided in Table S1. Through preliminary trials, the box settings were chosen by centering on the original position of the co-crystalized ligand and then optimized to achieve an RMSD of <2 Å in the redocked co-crystalized ligand. The 3D diagrams of protein-peptide docking models were visualized with BIOVIA Discovery Studio Visualizer. The 2D diagrams of protein-peptide interaction were prepared with LigPlot+ v.2.2 [23,24].

#### 2.6.4. Prediction of Physicochemical Properties, Toxicity, Allergenicity, and Cell-Penetrating Potential

Physicochemical properties of peptides were predicted by using the Peptides Package in R (https://rdrr.io/snippets/) [30] (access date: 26 June 2021). The online tool ToxinPred (https://webs.iiitd.edu.in/raghava/toxinpred/index.html) (access date: 24 June to 5 July 2021) was used to predict the toxicity of selected peptides. The prediction was done based on a support vector machine with a default threshold of 0.0 [31]. Subsequently, AllerTOP v.2.0 (https://www.ddg-pharmfac.net/AllerTOP) (access date: 24 June to 5 July 2021) was used for allergenicity prediction [32]. Prediction of cell-penetrating potential was performed with BChemRF-CPPred (http://comptools.linc.ufpa.br/BChemRF-CPPred/) (access date: 24 June to 5 July 2021), by using options FC-3 and Model Version 2.0 in the setting [33].

### 2.7. Statistical Analysis

Data were presented as mean ± standard errors (*n* = 3). Statistical analysis was performed by using the SAS University Edition Software (Version 9.4, SAS Institute, Cary, NC, USA). The data were first analyzed by using the one-way ANOVA test. Subsequent multiple comparisons of the means were done by using Tukey’s test at a 95% confidence interval (*p* < 0.05).

## 3. Results and Discussion

### 3.1. Purification of T1H by UF

**Figure 1:**
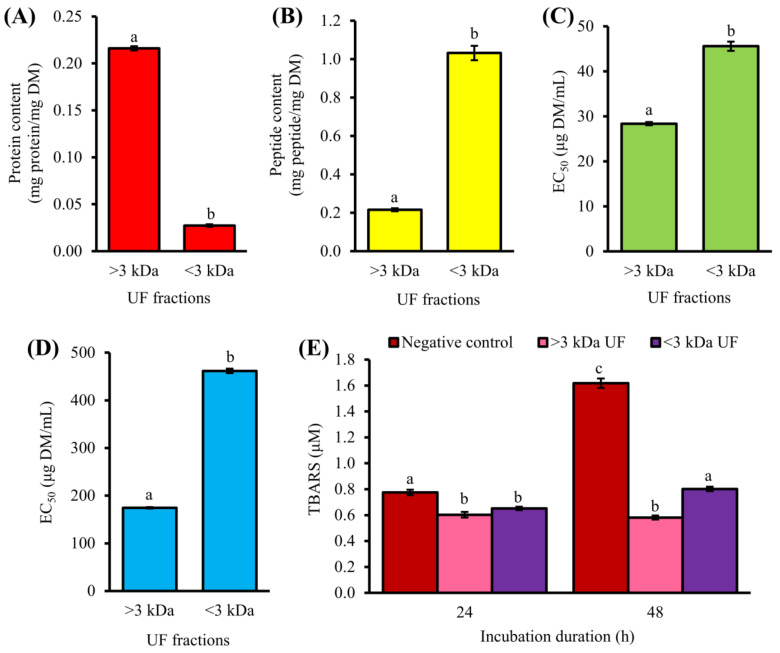
(**A**) Protein content, (**B**) peptide content, (**C**) 2,2′-azino-bis(3-ethylbenzothiazoline-6-sulfonic acid) diammonium salt radical cation (ABTS^•+^) scavenging activity and (**D**) hydrogen peroxide (H_2_O_2_) scavenging activity of ultrafiltration (UF) fractions. (**E**) Thiobarbituric acid reactive species (TBARS) values of negative control and UF fractions tested at 0.5 mg dry mass (DM)/mL. For each bar chart, data are mean ± standard errors (*n* = 3). Mean values denoted by different superscript letters are significantly different (*p* < 0.05) according to Tukey’s test.

T1H was separated by UF, yielding two UF fractions. The >3 kDa fraction exhibited stronger antioxidant activity than the <3 kDa fraction based on their EC_50_ for ABTS^•+^ (28.4 and 45.6 µg dry mass (DM)/mL, respectively) and H_2_O_2_ (174.5 and 461.7 µg DM/mL, respectively) scavenging activities (Figure 1C,D). TBARS value of the negative control increased from 0.7 to 1.6 µM MDA equivalents after incubation of the lecithin liposomes from 24 to 48 h, indicating an increase in lipid peroxidation. As revealed by the TBARS values, treatment with 0.5 mg DM/mL of >3 kDa and <3 kDa fractions inhibited lipid peroxidation by 22% and 16% after 24 h, and by 64% and 50% after 48 h, respectively (Figure 1E). Our results are concordant with previous observations on the antioxidant activities of the UF fractions of fennel seed hydrolysate [34]. In theirs and this study, the superiority of the >3 kDa fraction in scavenging radicals relative to the <3 kDa fraction was observed. This may be attributed, in part, to the presence of large compounds, such as long peptides, partially degraded proteins, or other components with antioxidant properties in the >3 kDa fraction. In fact, the protein content of the >3 kDa fraction was 8-fold higher than that of the <3 kDa fraction (Figure 1A). By contrast, the <3 kDa fraction was peptide-rich, with peptide content 5-fold greater than the >3 kDa fraction (Figure 1B).

Notably, the <3 kDa fraction was comparable or superior to T1H in scavenging ABTS^•+^ and repressing lipid peroxidation, based on activity data reported in our previous study [4]. This implies that at least part of the antioxidant activities exhibited by T1H could be attributed to the presence of antioxidant peptides in the hydrolysate. The <3 kDa fraction was weaker than antioxidant tripeptide GSH (EC_50_ 6.7 µg DM/mL) as an ABTS^•+^ scavenger but 3-fold stronger than GSH (EC_50_ 1378.7 µg DM/mL) as H_2_O_2_ scavenger. H_2_O_2_ is an ROS molecule that can diffuse through biological membranes and convert to highly reactive hydroxyl radicals in the body cells [5]. Hence, the <3 kDa UF fraction likely contained antioxidant peptides with the ability to scavenge biologically relevant ROS. In this study, a time-dependent increase in the lipid peroxidation inhibitory activity of the UF fractions was observed (Figure 1E). This observation agrees with a previous study that compared the ability of corn gluten meal hydrolysates to inhibit lipid peroxidation in a ground pork system following 8 h and 16 h of treatment [35]. Our result showed that the lipid peroxidation inhibitory effects of the CS peptides could persist up to 48 h. Low molecular weight (MW) peptides are desirable because they could be more easily absorbed by the body compared to high MW peptides [5]. Thus, the peptide-enriched <3 kDa fraction was selected for further purification.

### 3.2. Purification of <3 kDa Fraction by GFC

**Figure 2:**
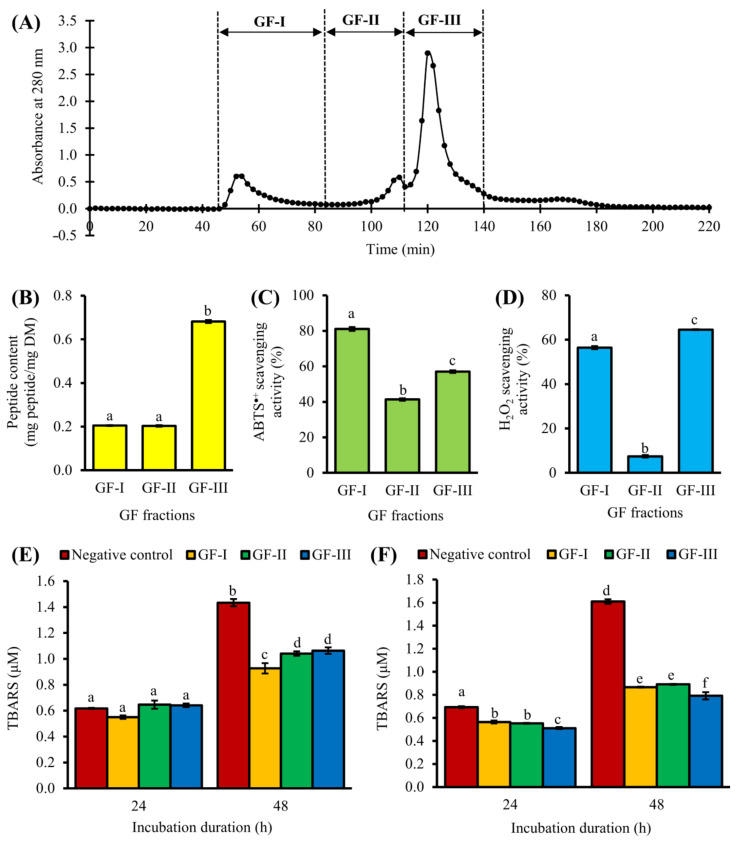
Purification of the <3 kDa fraction by gel filtration chromatography (GFC). (**A**) Elution profile, (**B**) peptide content, (**C**) ABTS^•+^ scavenging activity (at 30 µg peptide/mL), and (**D**) H_2_O_2_ scavenging activity (at 150 µg peptide/mL) of pooled GFC fractions. TBARS values of negative control and GFC fractions (**E**) at 0.1 mg peptide/mL and (**F**) at 0.5 mg DM/mL. Data are mean ± standard errors (*n* = 3). For each bar chart, mean values denoted by different superscript letters are significantly different (*p* < 0.05) according to Tukey’s test.

Purification by GFC resulted in three pooled fractions: GF-I, GF-II, and GF-III (Figure 2A). Among the three, GF-I exhibited the strongest effects in ABTS^•+^ scavenging activity (Figure 2C). GF-I possibly comprised more non-aromatic peptide residues with radical scavenging activity, such as Leu and Pro [36] than the other two fractions. Meanwhile, GF-III showed the highest H_2_O_2_ scavenging activity among the three pooled fractions (Figure 2D). All three pooled GFC fractions could dampen the time-dependent increase in lipid peroxidation in the liposome model at 0.1 mg peptide/mL, with 26–35% inhibition of TBARS formation after 48 h (Figure 2E), although their activities were significantly lower than that of GSH (57% inhibition after 48 h). The peptide content of GF-III (0.68 mg peptide/mg DM) was 3.5-fold greater than those of GF-I and GF-II (Figure 2B). When compared to GF-III, 3.5-fold higher DM of GF-I and GF-II was required to achieve the standardized peptide concentration used for evaluating the lipid peroxidation inhibitory activity depicted in Figure 2E. It can be anticipated that when expressed in terms of DM, the lipid peroxidation inhibitory activity of GF-III may exceed that of GF-I. Thus, we also analyzed the lipid peroxidation inhibitory activity of the three pooled fractions at 0.5 mg DM/mL. As expected, among the three fractions, GF-III showed the strongest inhibition of TBARS formation, with 26% and 51% inhibition after 24 and 48 h, respectively (Figure 2F). The antioxidant activity of GF-III could be owing to its relatively high absorbance at 280 nm (Figure 2A), which suggests an abundance of aromatic amino acid residues (e.g., Phe, Tyr, and Trp) in the pooled fraction. In keeping with this study, a previous study on Chinese chestnut also found that the GFC fraction with the most prominent absorbance at 280 nm had the highest antioxidant activity among all GFC fractions [37]. 

In our GFC experiment, GF-II (intermediate molecular size) had the lowest antioxidant activity, whereas GF-I (greatest molecular size) and GF-III (smallest molecular size) had relatively higher antioxidant activities. Hence, our results suggest that the antioxidant potential of the GFC fractions is not directly related to their molecular size. This is in agreement with the lack of explicit relationship between antioxidant activity and molecular size among 81 corn gluten meal peptide fractions collected in a GFC experiment [38]. On the other hand, peptide content data and the different trends in the lipid peroxidation inhibitory activities of the pooled fractions when tested based on DM and peptide mass pointed to the presence of non-peptide constituents in the GFC fractions. Thus, further purification was desirable. Considering that GF-III was the richest in peptide content and to discover peptides containing aromatic amino acid residues from T1H, we proceeded to perform purification on GF-III. 

### 3.3. Purification of GF-III by SCX-SPE

**Figure 3:**
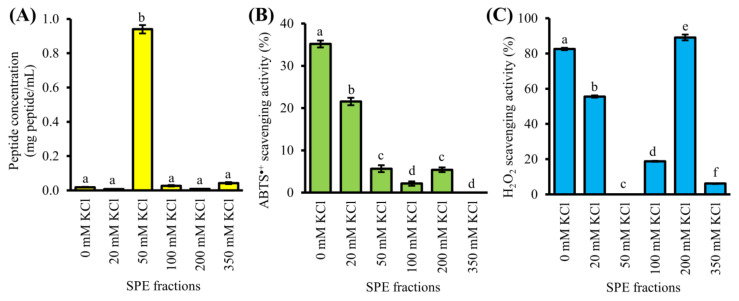
Purification of the GF-III fraction by strong-cation-exchange solid-phase extraction (SPE). (**A**) Peptide concentration, (**B**) ABTS^•+^ scavenging activity (at 0.7 µg peptide/mL), and (**C**) H_2_O_2_ scavenging activity (at 10 µg peptide/mL) of SPE fractions. Data are mean ± standard errors (*n* = 3). For each bar chart, mean values denoted by different superscript letters are significantly different (*p* < 0.05) according to Tukey’s test.

GF-III was further purified by SCX-SPE, producing six SPE fractions. As shown in Figure 3A, most of the peptide constituents of GF-III were found in 50 mM KCl fraction (0.9 mg peptide/mL), which was 22–118 times greater than the other five SPE fractions. The 50 mM KCl fraction showed relatively low or no ABTS^•+^ and H_2_O_2_ scavenging activities at the peptide concentrations tested (Figure 3B,C). Our result suggests that SCX-SPE has partitioned most of the non-antioxidant peptides and/or peptides with weak antioxidant activity into the 50 mM KCl fraction.

The effectiveness of SCX-SPE in concentrating the antioxidant peptides into single fractions were also evident, as affirmed by the enhancement in antioxidant activity after the SPE step. Briefly, 30 µg peptide/mL of GF-III scavenged 57% of ABTS^•+^ (Figure 2C). After the purification of GF-III by SCX-SPE, the resultant 0 and 20 mM KCl fractions scavenged 35% and 22% ABTS^•+^ at a 43-fold lower concentration (0.7 µg peptide/mL), respectively (Figure 3B). By estimation, the 0 and 20 mM KCl fractions may be 26-fold and 17-fold stronger than GF-III as ABTS^•+^ scavengers, respectively. Other studies also showed 33% [39] and 44% [34] improvement in the ABTS^•+^ scavenging activity of a peptide fraction purified by the SCX-SPE. The potency of the 0 and 20 mM KCl fractions over the other SPE fractions as ABTS^•+^ scavenger may be attributed to the presence of negatively charged amino acids (e.g., Glu) [40] or proton-donating amino acids (e.g., Trp and Gln) [38]. The presence of such residues may impart antioxidant activity to peptides by transferring electrons or protons to free radicals [36]. For instance, rapeseed peptides predominantly made up of Glu (19.5%) were reported to have potent radical scavenging activity [40]. Corn gluten meal-derived peptides made up of 67% of Trp and Gln displayed high ABTS^•+^ scavenging activity [38].

A similar improvement in the antioxidant activity of SPE fractions following SCX-SPE was revealed by the H_2_O_2_ scavenging assay. For example, 150 µg peptide/mL of GF-III scavenged 65% H_2_O_2_ (Figure 2D). In contrast, at a 15-fold lower concentration (10 µg peptide/mL), the 0 and 200 mM KCl fractions scavenged more than 80% of H_2_O_2_ (Figure 3C). The 20 mM KCl fraction also scavenged 56% H_2_O_2_ when tested at 10 µg peptide/mL. Thus, based on theoretical calculations, our results imply a 13–21-fold improvement in the H_2_O_2_ scavenging activity of the 0, 20, and 200 mM KCl fractions resulting from the purification of GF-III by using SCX-SPE. Altogether, the 0, 20, and 200 mM KCl fractions potentially contained potent antioxidant peptides; thus, they were taken to peptide sequencing.

### 3.4. Identification and Characterization of Antioxidant Peptides

LC-MS/MS analysis identified 29 peptide sequences comprising 6–14 residues (633.33 to 1517.81 Da) from the 0, 20, and 200 mM KCl fractions (Table 1). This range of peptide masses agrees with the observation that the molecular masses of food-derived antioxidant peptides commonly range between 500–1800 Da [36]. Twenty-three of the 29 peptides contain 11–56% aliphatic amino acid residues (Table 1). Such residues are responsible for the thermal stability of proteins [30]. Two thermal-stable antioxidant peptides WAFAPA and MYPGLA that were identified from the blue-spotted stingray, for instance, are composed of 50% and 33% of aliphatic residues, respectively [11]. Based on the comparison of the aliphatic index, 10 of the 29 peptides were likely superior to both WAFAPA and MYPGLA in terms of thermal stability (Table 1). The discovery of such peptides also supports our previous observation of the thermal stability of T1H, the protein hydrolysate from which the 29 peptides were purified. T1H retained its radical scavenging and ferric reducing activity at temperatures up to 100 °C [4]. These CS peptides that are likely to be thermal-stable can thus be utilized as alternatives for food additives to address the concerns regarding the food processing heat treatment.

Amino acid composition is a key factor influencing the antioxidant activity of peptides [5]. Hydrophobic residues made up 33–86% of the compositions of the 29 CS peptides (Table 1). The presence of hydrophobic residue-containing peptides may account for the lipid peroxidation inhibitory effect of T1H [4], as well as that of <3 kDa UF (Figure 1E) and GF-III (Figure 2E,F), the peptide fractions which the 29 peptides were purified from. Hydrophobic residues may enhance the interaction of antioxidant peptides with lipid-soluble free radicals, thus attenuating the progression of lipid peroxidation [5]. Lipid oxidation is one of the major contributors to the deterioration of food quality during food processing and storage [5]. Thus, these CS peptides, as well the CS hydrolysate and partially purified fractions containing them, may be useful in the preservation of lipid-rich foods. On the other hand, it has been reported that peptides with C-terminal Lys could act as potent H_2_O_2_ scavengers [41]. Thus, MAPRTPRK and THAVKGVVHK in the 0 mM KCl fraction, FMFFVYK and MCFHHHFHK in the 20 mM KCl fraction, as well as DFPGAK, AGFPLGK, and AMQQDK in the 200 mM KCl fraction may have contributed to the H_2_O_2_ scavenging activity of the three SPE fractions. 

The 0 mM KCl fraction had the highest proportion of peptides containing aromatic residues (9 peptides), followed by the 200 mM KCl (4 peptides) and the 20 mM KCl fractions (3 peptides). Aromatic residues may promote the antioxidant activity of peptides by donating protons to the electron-deficient radicals [36]. Notably, the 0 mM KCl fraction had the highest number of basic residue-containing peptides and the highest percentage of basic residues in peptides (Table 1). Thus, the strongest ABTS^•+^ scavenging activity of the 0 mM KCl fraction may be attributed to its richness in peptides comprising aromatic and basic amino acids. Our result agrees with the finding that abundance in basic amino acids may account for the strong ABTS^•+^ scavenging activity of a Chinese chestnut peptide fraction [37].

As revealed by the AnOxPePred analysis, 10 CS peptides (MCFHHHFHK, NLEGYR, AGFPLGK, FMFFVYK, NMVPGR, PVVWAAKR, DFPGAK, FSCPLVMKGPNGLR, RHGSGR, and VHFNKGKKR) had comparable or higher free radical scavenger (FRS) scores relative to the four reference peptides VGPWQK, MYPGLA, FPLPSF and WAFAPA (Table 2). The four reference peptides were empirically proven as ABTS^•+^ scavengers [11,39,42]. Our results are in accordance with the finding that His, Trp, Tyr, and Pro are common in free-radical-scavenging peptides [17]. In this study, the four amino acids account for 11–44% of the residues making up the 10 CS peptides and the four reference peptides (Table 2). Notably, His-containing peptides were only found in the 0 mM KCl (THAVKGVVHK and VHFNKGKKR) and 20 mM KCl (RHGSGR and MCFHHHFHK) fractions, but none in the 200 mM KCl fraction (Table 1). This is in keeping with our observation that the 0 and 20 mM KCl fractions had at least 4-fold greater ABTS^•+^ scavenging activity than the 200 mM KCl (Figure 3B). Our results, therefore, support the role of His residues in imparting radical scavenging activity to peptides [36]. 

### 3.5. Molecular Docking between CS Peptides and ABTS^•+^

Docking simulation was performed to clarify the interactions between ABTS^•+^ and the 10 CS peptides with the best FRS scores. All seven peptides originating from the 0 and 20 mM KCl fractions had higher binding affinities towards ABTS^•+^ than the two peptides (AGFPLGK and DFPGAK) from the 200 mM KCl fraction (Table 3). Five of the seven peptides (from 0 and 20 mM KCl fractions) were also stronger than NLEGYR (from 200 mM KCl fraction) in binding to ABTS^•+^. The overall trend is in accordance with the relative levels of in vitro ABTS^•+^ scavenging activity of the three SPE fractions (Figure 3B). Notably, the binding affinities of all seven peptides from the 0 and 20 mM KCl fractions were more negative than that of reference peptides MYPGLA. The binding energy of three peptides MCFHHHFHK, VHFNKGKKR, and PVVWAAKR was up to 21% more negative than all four reference peptides (Table 3). Taken together, the seven peptides originating from the 0 and 20 mM KCl fractions could bind to ABTS^•+^ similarly or more stably than could the four reference peptides. Peptides that bind stably to free radicals can neutralize them. For instance, FPLPSF that was predicted to bind to ABTS^•+^ has been experimentally demonstrated to quench ABTS^•+^ in vitro [42]. Furthermore, our prediction of WAFAPA binding to ABTS^•+^ more stably than could MYPGLA (Table 3) is also consistent with their relative in vitro antioxidant activity [11]. Altogether, our results suggest that the stronger ABTS^•+^ scavenging activities of the 0 and 20 mM KCl fractions, relative to that of the 200 mM KCl fraction, could be accounted for, at least in part, by the affinity of their seven peptides to ABTS^•+^.

Our LigPlot+ analysis indicates significant participation of aromatic residues in CS peptide-ABTS^•+^ interactions (Table 3). For instance, aromatic residues in 67% of the aromatic residue-containing peptides could bind to ABTS^•+^ through hydrophobic interactions. Aromatic residue-ABTS^•+^ interactions made up 14–100% of the total number of interactions between individual aromatic residue-containing peptides and ABTS^•+^. Remarkably, all interactions formed between the best-binding-affinity MCFHHHFHK and ABTS^•+^ were contributed by the aromatic residues Phe and His (Table 3). Four CS peptides, namely VHFNKGKKR, PVVWAAKR, FSCPLVMKGPNGLR, and NMVPGR, were predicted to have the highest number of interactions with ABTS^•+^. Notwithstanding, the lack of participation of aromatic residues in the interactions between ABTS^•+^ and the two peptides FSCPLVMKGPNGLR and NMVPGR apparently made their binding to ABTS^•+^ 12–15% less stable relative to VHFNKGKKR and PVVWAAKR. Similarly, both WAFAPA and MYPGLA were predicted to form comparable numbers of interactions with ABTS^•+^. However, the lack of participation of aromatic residues in MYPGLA-ABTS^•+^ interaction may explain the reported weaker ABTS^•+^ scavenging activity of MYPGLA when compared with WAFAPA [11]. Moreover, we also observed the significant participation of basic residues in peptide-ABTS^•+^ interactions. Briefly, basic residues in 90% of basic residue-containing CS peptides were involved in the interactions with ABTS^•+^. Such interactions account for 33–80% of the total number of interactions between individual basic residue-containing peptides and ABTS^•+^. To further verify the role of the basic residues of peptides in binding to ABTS^•+^, in silico alanine substitution was performed on those that were involved in ABTS^•+^-peptide interactions, followed by docking of the alanine-substituted peptides to ABTS^•+^. The binding affinities of all CS peptides, except FSCPLVMKGPNGLR, were diminished upon alanine mutagenesis of selected basic residues in the peptides (Table 3 and Table 4). For instance, alanine substitution of His6 in MCFHHHFHK has resulted in a 15% reduction in the binding affinity of peptides towards ABTS^•+^. Besides, a decline of 24% in binding affinity of NMVPGR towards ABTS^•+^ was observed upon alanine substitution of Arg6. Our results suggest that the basic residues are likely to be critical in binding and stabilizing ABTS^•+^. This observation further reinforces our finding that the 0 mM KCl fraction with the highest number of basic residue-containing peptides displayed the strongest ABTS^•+^ scavenging activity (Table 1 and Figure 3B). Besides, Leu-ABTS^•+^ interactions were observed in 67% of Leu-containing CS peptides (Table 3). Our observation agrees with a previous report of the participation of Leu in the binding between antioxidant peptides and ABTS^•+^ [42].

### 3.6. Molecular Docking of Peptides on Keap1

Food-derived bioactive peptides, in addition to scavenging free radicals, can confer cellular protection by modulating the gene expression and activities of antioxidant and oxidant enzymes [5]. Given this, we conducted a docking-based screening experiment to unravel the potential of the 29 CS peptides identified in this study in interacting with cellular protein targets that can regulate the endogenous oxidant status: Keap1, MPO, and XO. Soy-derived DEQIPSHPPR was predicted in molecular docking study to interact stably with Keap1, in keeping with its demonstrated ability to disrupt Keap1-Nrf2 binding and increase Nrf2 levels in the nucleus [6]. Hence, DEQIPSHPPR was used as a reference peptide for comparison with CS peptides. Our docking results show that 13 of the 29 CS peptides could bind to Keap1 similarly or more stably than DEQIPSHPPR (Table S2). Further in silico screening for low toxicity and allergenicity as well as high cell-penetrating potential narrowed down the 13 CS peptides to five, namely NDGPSR, NLEGYR, NMVPGR, SSPATGGSLR, and NANSLAGPQR (Table 5). Screening based on these parameters allows the search for CS peptides that might be able to cross the cell membrane barrier and block the Keap1-Nrf2 interaction in cells with minimal or no harmful effects. Unlike the five CS peptides, the reference peptide DEQIPSHPPR may elicit allergy (Table 5), hence it is less desirable for the application of functional food ingredients.

The five aforementioned CS peptides successfully docked into Keap1 and interacted with 3–6 of the seven key residues known to be involved in Keap1-Nrf2 interactions (Table 6). Our analysis on the reference peptide DEQIPSHPPR agrees with a previous report of its interaction with the key residues Arg380, Asn382, and Arg415 of Keap1 [6]. The participation of the three residues was also observed in the interactions between Keap1 and CS peptides (NANSLAGPQR and SSPATGGSLR) (Table 6). Besides, the binding of NLEGYR to Keap1 via hydrogen bond, hydrophobic interaction, and the salt bridge was also found in the DEQIPSHPPR-Keap1 interaction (Table 6). Thus, the five CS peptides apparently have comparable Keap1-binding properties as DEQIPSHPPR and could potentially activate the Keap1/Nrf2 pathway, triggering cellular antioxidant defense.

### 3.7. Molecular Docking of Peptides on MPO

In this study, CS peptides were compared with the soy tripeptide VPY and false abalone-derived DTETGVPT in their affinity towards MPO. Oral administration of VPY was shown to induce a 4-fold reduction in the MPO activity of mice [43]. Meanwhile, DTETGVPT is a potential inhibitor of MPO, as revealed by molecular docking simulation [44]. Our results revealed that 12 of the 29 CS peptides had up to 23% stronger affinity to MPO when compared with DTETGVPT, but were all weaker than VPY (Table S2). Five of the 12 potential MPO-binding CS peptides, namely NDGPSR, NLEGYR, NMVPGR, KRYFKR, and RHGSGR, were found to have low toxicity, low allergenicity, and cell-penetrating potential (Table 5). The five CS peptides would thus have an advantage over the reference peptide VPY (probable allergen) and DTETGVPT (non-cell-penetrating peptide) (Table 5) in the context of the application as food additives or functional food ingredients.

The interactions between the five CS peptides and MPO active site residues were highly similar to those between the reference peptides (VPY and DTETGVPT) and MPO (Table 7). With the exemption of NLEGYR, CS peptides and reference peptides were all forming only the hydrophobic interaction with the key residues in MPO active site. Likewise, similar to the reference peptides, each CS peptide could interact with 4–5 of the seven key residues in the active site of MPO. These key residues contributed to the stability of the interaction between 7-benzyl-1*H*-[1,2,3]triazolo[4,5-b]pyridin-5-amine, the bound inhibitor in the MPO crystal, and the active site of MPO [25]. As observed in the two reference peptides, all the five CS peptides could bind directly to the heme moiety of MPO (Table 7). Blockage of the heme pocket of MPO could preclude the access of substrates to the MPO active site, suppressing the enzyme’s action-driving ROS production [45].

### 3.8. Molecular Docking of Peptides on XO

Only 14 of the 29 CS peptides were docked onto XO successfully, yielding negative values for binding affinity (Table S2). Docking of NDGPSR and DFPGAK onto XO involved the lowest binding energy, thus the strongest affinity, among the 14 peptides. Furthermore, NDGPSR and DFPGAK were predicted to bind equally stably to XO as was ACECD, the reference peptide. ACECD, an XO inhibitory peptide derived from Skipjack tuna hydrolysate, was reported to exert its inhibition by binding to the active site of XO [46]. In light of the predicted allergenicity and non-cell-penetrating potential of DFPGAK (Table 5), we proceeded to analyze the intermolecular interactions in only the NDGPSR-XO docked model.

As shown in Table 8, our LigPlot+ analysis revealed that NDGPSR could interact with catalytically critical residues (Glu802 and Arg880), substrate binding-residue (Phe914, Phe1009, and Thr1010), and the residues associated with the extended solvent-accessible channel leading to the molybdenum active center (Leu873, Val1011, Phe1013, and Leu1014) [26]. Most of such interactions are hydrophobic in nature. The dominance of hydrophobic interactions was also observed in our ACECD-XO docked model (Table 8). Meanwhile, NDGPSR could form hydrophobic interaction with Glu1261, an amino acid around the active center of XO, and contribute to the catalytic reaction of XO [46]. Moreover, as observed in ACECD, NDGPSR could also interact with Leu648 and Phe649 through hydrophobic interactions. The two residues are at the gate of the aforementioned solvent-accessible channel [26]. Interactions with Leu648 and Phe649 were reported to enhance the potency of quercetin as an XO inhibitor [26]. Altogether, our results suggest that NDGPSR can potentially occupy the catalytic center of XO, hindering the entry of XO substrates, thereby suppressing XO activity and ROS generation. In addition to their protective role in cells, antioxidant peptides with XO inhibitory activity are useful in reducing milk-fat oxidation in the dairy industry [9]. Thus, NDGPSR may also be developed into a potent antioxidant for the formulation of dairy products and other oxidation-prone foods.

**Figure 4:**
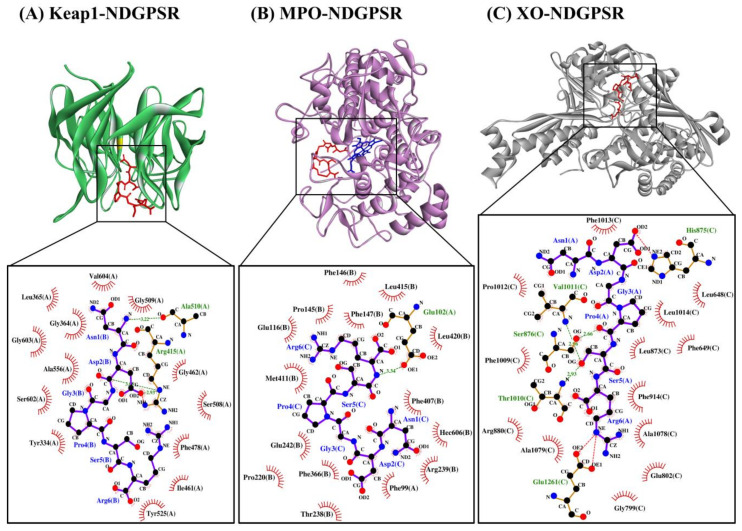
The docked models of NDGPSR interacting with (**A**) Keap1, (**B**) MPO, and (**C**) XO in 3D (top) and 2D (bottom) diagrams. In the 3D diagrams, NDGPSR is displayed in red; the heme moiety of MPO in (**B**) is displayed in blue. In the 2D diagrams, bonds of proteins are in orange, whereas those of peptides are in purple. Hydrophobic interactions, hydrogen bonds, and salt bridges are represented in red spoked arcs, green dashed lines, and red dashed lines, respectively.

Our in silico analysis revealed three multifunctional peptides that are also non-toxic, non-allergenic, and have cell-penetrating potential, namely NDGPSR, NLEGYR, and NMVPGR. Among the three, NDGPSR could be a potential inhibitor of Keap1-Nrf2 interaction, MPO, and XO. Models of NDGPSR docked to the three protein targets are shown in Figure 4. Moreover, NLEGYR and NMVPGR could be potential dual-function inhibitors of Keap1-Nrf2 interaction and MPO (Table S2). Antioxidant peptides possessing multiple functionalities likely have greater versatility and commercial value when compared to other antioxidant peptides [36]. In this context, the three multifunctional peptides, with their safety and cell-penetrating properties, are desirable candidates for the future development of functional food ingredients and/or health supplements.

## 4. Conclusions

In this study, 29 potential antioxidant peptides were purified and identified, for the first time, from a CS hydrolysate. The prevalence of aromatic and basic residues, in addition to binding affinity to ABTS^•+^, as revealed by molecular docking simulation, may account for the antioxidant activities of the peptides. Our in silico study also unraveled the potential of the peptides as inhibitors of Keap1-Nrf2 interaction, MPO and XO. NDGPSR stood out among the 29 peptides for its concurrent affinities towards the three protein targets, besides being predicted as non-toxic, non-allergenic, and having cell-penetrating potential. Taken together, our findings highlight the potential of CS as a source of antioxidant peptides with desirable properties for future applications in functional food and drug discovery. Future investigations by using in vitro and in vivo models are warranted for more in-depth exploration of CS-derived antioxidant peptides highlighted in this study.

## Figures and Tables

**Table 1:** Physicochemical properties of the peptides identified from the 0, 20, and 200 mM KCl fractions.

SPE Fractions	Peptides	Measured *m*/*z* [M + 2H]^2^	Molecular Mass (Da) ^a^	Aromatic Residues (%) ^b^	Basic Residues (%) ^b^	Hydrophobic Residues (%) ^b^	Aliphatic Residues (%) ^b^	Aliphatic Index ^b^
0 mM KCl	KRYFKR	449.28	896.57	33	67	33	0	0
	PRVRVAGR	455.79	909.58	0	38	63	38	85
	PVVWAAKR	463.79	925.57	13	25	75	50	98
	QVASGPLQR	478.28	954.55	0	11	56	33	87
	MAPRTPRK	478.78	955.57	0	38	50	13	13
	NKVVKLMR	494.31	986.62	0	38	50	38	121
	KVPLAVFSR	508.82	1015.64	11	22	67	44	119
	LKKGSPLKR	513.84	1025.69	0	44	44	22	87
	FQLKPVFR	517.82	1033.63	25	25	63	25	85
	THAVKGVVHK	538.34	1074.67	20	40	50	40	97
	YTWKFKGR	543.31	1084.61	38	38	50	0	0
	ARVPQQSYR	552.80	1103.61	11	22	44	22	43
	VHFNKGKKR	557.34	1112.69	22	56	33	11	32
	TAPLSSKALKR	586.37	1170.73	0	27	45	36	89
	FSCPLVMKGPNGLR	759.91	1517.81	7	14	71	21	76
20 mM KCl	RHGSGR	335.18	668.37	17	50	33	0	0
	NMVPGR	337.17	672.34	0	17	67	17	48
	FMFFVYK	491.25	980.50	57	14	86	14	41
	MCFHHHFHK	612.27	1222.53	67	56	44	0	0
200 mM KCl	DFPGAK	317.66	633.33	17	17	67	17	17
	NDGPSR	323.15	644.29	0	17	33	0	0
	AGFPLGK	345.20	688.41	14	14	86	29	70
	AMQQDK	360.66	719.32	0	17	33	17	17
	NLEGYR	376.19	750.38	17	17	50	17	65
	YETLNR	398.20	794.41	17	17	33	17	65
	MPPKSTR	408.72	815.43	0	29	43	0	0
	TAGASLVAR	423.25	844.49	0	11	67	56	109
	SSPATGGSLR	466.74	931.49	0	10	50	20	49
	NANSLAGPQR	514.27	1026.55	0	10	50	30	59

^a^ Molecular mass was calculated from the *m*/*z* value determined by liquid chromatography-tandem mass spectrometry. ^b^ Percentages of aromatic, basic, hydrophobic, and aliphatic residues as well as aliphatic index were computed by using Peptides Package in R. Aliphatic indices of reference peptides WAFAPA and MYPGLA as predicted by Peptides Package in R were 50 and 82, respectively.

**Table 2:** Free radical scavenger (FRS) scores of corn silk peptides were identified from the three SPE fractions, in comparison with reference peptides.

Peptides	SPE Fractions	FRS Scores
MCFHHHFHK	20 mM KCl	0.68068
VGPWQK *	-	0.52254
MYPGLA *	-	0.49386
NLEGYR	200 mM KCl	0.48158
AGFPLGK	200 mM KCl	0.44866
FMFFVYK	20 mM KCl	0.44397
NMVPGR	20 mM KCl	0.44319
PVVWAAKR	0 mM KCl	0.43744
DFPGAK	200 mM KCl	0.43574
FPLPSF *	-	0.43352
FSCPLVMKGPNGLR	0 mM KCl	0.41864
WAFAPA *	-	0.41519
RHGSGR	20 mM KCl	0.41088
VHFNKGKKR	0 mM KCl	0.41055
NANSLAGPQR	200 mM KCl	0.40415
QVASGPLQR	0 mM KCl	0.40213
MAPRTPRK	0 mM KCl	0.39973
NDGPSR	200 mM KCl	0.38760
KRYFKR	0 mM KCl	0.38352
YETLNR	200 mM KCl	0.37938
FQLKPVFR	0 mM KCl	0.37599
ARVPQQSYR	0 mM KCl	0.37580
YTWKFKGR	0 mM KCl	0.36769
AMQQDK	200 mM KCl	0.36324
SSPATGGSLR	200 mM KCl	0.35382
THAVKGVVHK	0 mM KCl	0.35200
MPPKSTR	200 mM KCl	0.33529
LKKGSPLKR	0 mM KCl	0.32957
PRVRVAGR	0 mM KCl	0.32698
KVPLAVFSR	0 mM KCl	0.32525
TAGASLVAR	200 mM KCl	0.32285
TAPLSSKALKR	0 mM KCl	0.29320
NKVVKLMR	0 mM KCl	0.27437

* Indicates reference peptides.

**Table 3:** Binding affinities and types of interactions between 10 corn silk peptides and ABTS^•+^, in comparison with four reference peptides.

Peptides	SPE Fractions	Binding Affinity (kcal/mol)	Peptide Residues Interacting with ABTS^•+ a^	
			Hydrogen Bond	Hydrophobic Interaction
MCFHHHFHK	20 mM KCl	−4.8	-	Phe3, His6, Phe7
VHFNKGKKR	0 mM KCl	−4.7	Lys7, Arg9	Val1, His2, Gly6, Lys7, Arg9
PVVWAAKR	0 mM KCl	−4.7	Arg8 (2)	Val2, Trp4, Ala5, Ala6, Arg8
FMFFVYK	20 mM KCl	−4.4	Lys7	Phe1, Phe3, Phe4, Lys7
FSCPLVMKGPNGLR	0 mM KCl	−4.2	Arg14 (2)	Leu5, Lys8, Gly9, Pro10, Gly12, Arg14
NMVPGR	20 mM KCl	−4.1	Asn1, Arg6 (2)	Asn1, Pro4, Gly5, Arg6
NLEGYR	200 mM KCl	−4.1	-	Tyr5, Arg6
RHGSGR	20 mM KCl	−3.9	Arg1, Arg6	Arg1, Gly5, Arg6
AGFPLGK	200 mM KCl	−3.7	-	Phe3, Pro4, Leu5
DFPGAK	200 mM KCl	−3.6	-	Pro3, Gly4, Lys6
FPLPSF *	-	−4.6	Phe1, Ser5	Phe1, Pro2, Leu3, Pro4, Ser5
WAFAPA *	-	−4.3	-	Trp1, Ala4, Pro5
VGPWQK *	-	−3.9	-	Pro3, Trp4, Lys6
MYPGLA *	-	−3.8	Pro3	Pro3, Leu5, Ala6

* Indicates reference peptides. ^a^ Number in brackets indicates the number of interactions.

**Table 4:** Binding affinities of corn silk peptides toward ABTS^•+^ upon alanine substitution of the basic residues that were involved in ABTS^•+^-peptide interactions.

Peptides ^a^	Basic Residues	Mutant Peptides	Binding Affinity (kcal/mol)
MCFHHHFHK	His6	MCFHHAFHK	−4.1
VHFNKGKKR	His2	VAFNKGKKR	−4.8
	Lys7	VHFNKGAKR	−5.0
	Arg9	VHFNKGKKA	−4.4
PVVWAAKR	Arg8	PVVWAAKA	−4.3
FMFFVYK	Lys7	FMFFVYA	−4.3
FSCPLVMKGPNGLR	Lys8	FSCPLVMAGPNGLR	−4.2
	Arg14	FSCPLVMKGPNGLA	−4.7
NMVPGR	Arg6	NMVPGA	−3.1
NLEGYR	Arg6	NLEGYA	−3.7
RHGSGR	Arg1	AHGSGR	−3.8
	Arg6	RHGSGA	−4.2
DFPGAK	Lys6	DFPGAA	−3.4

^a^ Peptides are arranged in the same order as in Table 3.

**Table 5:** Toxicity, allergenicity, and cell-penetrating ability predicted for selected corn silk peptides that have the same or higher affinity to Kelch-like ECH-associated protein 1, myeloperoxidase, and xanthine oxidase, in comparison with reference peptides.

Peptides	Toxicity	Allergenicity	CPP Prediction
NDGPSR	Non-toxin	Probable non-allergen	CPP
NLEGYR	Non-toxin	Probable non-allergen	CPP
NMVPGR	Non-toxin	Probable non-allergen	CPP
SSPATGGSLR	Non-toxin	Probable non-allergen	CPP
NANSLAGPQR	Non-toxin	Probable non-allergen	CPP
KRYFKR	Non-toxin	Probable non-allergen	CPP
RHGSGR	Non-toxin	Probable non-allergen	CPP
YETLNR	Non-toxin	Probable non-allergen	Non-CPP
AGFPLGK	Non-toxin	Probable non-allergen	Non-CPP
KVPLAVFSR	Non-toxin	Probable non-allergen	Non-CPP
TAGASLVAR	Non-toxin	Probable allergen	Non-CPP
YTWKFKGR	Non-toxin	Probable allergen	CPP
AMQQDK	Non-toxin	Probable allergen	CPP
MPPKSTR	Non-toxin	Probable allergen	CPP
PVVWAAKR	Non-toxin	Probable allergen	CPP
DFPGAK	Non-toxin	Probable allergen	Non-CPP
FMFFVYK	Non-toxin	Probable allergen	Non-CPP
QVASGPLQR	Non-toxin	Probable allergen	Non-CPP
DEQIPSHPPR *	Non-toxin	Probable allergen	Non-CPP
DTETGVPT *	Non-toxin	Probable non-allergen	Non-CPP
VPY *	Non-toxin	Probable allergen	CPP
ACECD *	Non-toxin	Probable allergen	CPP

* Indicates reference peptides. CPP, cell-penetrating peptide.

**Table 6:** Binding affinities and types of interactions between Kelch-like ECH-associated protein 1 (Keap1) and five corn silk peptides predicted as non-toxic, non-allergenic and cell-penetrating peptides, in comparison with a reference peptide.

Peptides	Binding Affinity (kcal/mol)	Interaction with Keap1 ^a^		
		Hydrogen Bond	Hydrophobic Interaction	Salt Bridge
NLEGYR	−8.7	**Arg415**, **Arg483**, Ser508, Gln530, Ser555	**Tyr334**, Ser363, Gly364, Leu365, Ala366, **Arg415**, Ile416, Gly417, Gly462, Phe478, **Arg483**, Ser508, Gly509, Ala510, Tyr525, Gln530, Ser555, Ala556, Leu557, **Tyr572**, **Phe577**, Ser602, Gly603, Val604	**Arg415**
NANSLAGPQR	−8.2	**Arg415** (3), Val418, Val465, **Arg483**	Ser363, Gly364, Leu365, **Arg380**, Asn382, Asn414, **Arg415**, Ile416, Gly417, Ile461, Gly462, Val463, Val465, Phe478, **Arg483**, Ser508, Gly509, **Tyr525**, Gln530, Ser555, Ala556, Ile559, **Phe577**, Gly603	-
NMVPGR	−8.1	Ser363, Leu365, Asn382, Ser602	**Tyr334**, Ser363, Gly364, Leu365, Ala366, Asn382, **Arg415**, Ile416, Ile461, Gly462, Ser508, Gly509, Ala510, **Tyr525**, Gln530, Ser555, Ala556, Ser602	-
SSPATGGSLR	−8.1	Ser363, **Arg380**, Asn414, **Arg415**, Ser431, Ser602	**Tyr334**, Gly364, Leu365, **Arg380**, Asn382, Asn414, **Arg415**, Ile416, Ser431, Gly433, His436, Gly462, Phe478, **Arg483**, Ser508, Gly509, Ala556, Ser602, Gly603	-
NDGPSR	−8.0	**Arg415** (2), Ala510	**Tyr334**, Gly364, Leu365, **Arg415**, Ile461, Gly462, Phe478, Ser508, Gly509, **Tyr525**, Ala556, Ser602, Gly603, Val604	-
DEQIPSHPPR *	−8.0	**Tyr334**, Asn414, **Arg415** (4), Ser431, **Arg483** (3), Ser555	**Tyr334**, Ser363, **Arg380**, Asn382, Asn414, **Arg415**, Ser431, Gly433, His436, Gly462, Phe478, **Arg483**, Ser508, Gly509, **Tyr525**, Ser555, Ala556, **Tyr572**, **Phe577**, Ser602	**Arg483** (2)

* Indicates reference peptide. ^a^ Number in brackets indicates the number of interactions. Residues in bold are key residues (Tyr334, Arg380, Arg415, Arg483, Tyr525, Tyr572, and Phe577) in the binding site of Keap1 for nuclear factor erythroid 2-related factor 2 [7].

**Table 7:** Binding affinities and types of interactions between myeloperoxidase (MPO) and five corn silk peptides predicted as non-toxic, non-allergenic and cell-penetrating peptides, in comparison with two reference peptides.

Peptides	Binding Affinity (kcal/mol)	Interaction with MPO ^a^		
		Hydrogen Bond	Hydrophobic Interaction	Salt Bridge
NMVPGR	−6.6	-	**Phe99**, Thr100, Glu102, Glu116, Pro145, Phe147, Leu216, Pro220, **Arg239**, Glu242, **Phe366**, **Phe407**, Met411, Arg424, Hec606	-
NLEGYR	−6.5	**His95**	**His95**, **Phe99**, Glu102, Glu116, Pro145, Phe146, Phe147, Pro220, Thr238, **Arg239**, Glu242, **Phe407**, Val410, Met411, Leu420, Hec606	-
NDGPSR	−6.3	Glu102	**Phe99**, Glu102, Glu116, Pro145, Phe146, Phe147, Pro220, Thr238, **Arg239**, Glu242, **Phe366**, **Phe407**, Met411, Leu415, Leu420, Hec606	-
RHGSGR	−6.2	Thr100, Thr238	**Phe99**, Thr100, Glu102, Pro145, Phe146, Phe147, Leu216, Pro220, Thr238, **Arg239**, Glu242, **Phe366**, **Phe407**, Met411, Leu415, Hec606	Glu102 (5)
KRYFKR	−5.5	Thr100, Thr238	**His95**, **Phe99**, Thr100, Glu102, Glu116, Pro145, Phe147, Pro220, Thr238, **Arg239**, Glu242, **Phe366**, **Phe407**, Val410, Met411, Leu415, Leu420, Hec606	Glu102 (2)
VPY *	−7.4	-	**His95**, **Phe99**, Thr100, Glu102, Pro220, Thr238, **Arg239**, Glu242, **Phe366**, Hec606	-
DTETGVPT *	−5.5	Thr238	**Phe99**, Thr100, Glu102, Phe147, Pro220, Thr238, **Arg239**, Glu242, **Phe366**, **Phe407**, Met411, Leu415, Leu420, Hec606	-

* Indicates reference peptides. ^a^ Number in brackets indicates the number of interactions. Residues in bold are key residues (Gln91, His95, Phe99, Arg239, His336, Phe366, and Phe407) in the active site of MPO [25].

**Table 8:** Binding affinity and types of interactions between xanthine oxidase (XO) and corn silk peptide NDGPSR, which was predicted to be a non-toxic, non-allergenic, and cell-penetrating peptide, in comparison with a reference peptide.

Peptides	Binding Affinity (kcal/mol)	Interaction with XO ^a^		
		Hydrogen Bond	Hydrophobic Interaction	Salt Bridge
NDGPSR	−5.2	Ser876, **Thr1010**, **Val1011**	Leu648, Phe649, Gly799, **Glu802**, **Leu873**, His875, Ser876, **Arg880**, **Phe914**, **Phe1009**, **Thr1010**, **Val1011**, Pro1012, **Phe1013**, **Leu1014**, Ala1078, Ala1079, Glu1261	His875, Glu1261 (2)
ACECD *	−5.2	His875, Ser876	Leu648, Phe649, **Glu802**, **Leu873**, His875, Ser876, Glu879, **Phe914**, **Phe1009**, **Thr1010**, **Val1011**, Pro1012, **Phe1013**, **Leu1014**	-

* Indicates reference peptide. ^a^ Number in brackets indicates the number of interactions. Residues in bold are key residues (Glu802, Leu873, Arg880, Phe914, Phe1009, Thr1010, Val1011, Phe1013, and Leu1014) in the active site of XO [26].

## Data Availability

Data is contained within the article and supplementary material.

## Supplementary Materials

The following are available online at https://www.mdpi.com/article/10.3390/antiox10111822/s1, Table S1: Coordinates of box center and box size for molecular docking with different targets by using Webina 1.0.2, and the root mean square deviation (RMSD) values obtained, Table S2: Binding affinities of 29 corn silk peptides docked onto Keap1, MPO and XO.
