# Supplementary material for: Purification, Identification and Characterization of Antioxidant Peptides from Corn Silk Tryptic Hydrolysate: An Integrated In Vitro-In Silico Approach
Source: Antioxidants (Basel). 2021 Nov 17;10(11):1822. doi: 10.3390/antiox10111822 (PMC8615004; doi:10.3390/antiox10111822)
Supplement: Supplementary file 1 [file antioxidants-10-01822-s001.zip › antioxidants-1428647-supplementary.pdf]

Supplementary file

Table S1: Coordinates of box center and box size for molecular docking with different targets by using Webina 1.0.2, and the root mean square deviation (RMSD) values obtained.

Targets	Box Center			Box Size (Å)			RMSD Values
	x	y	z	x	y	z	
ABTS•+	28	28	28	25	25	25	-
Keap1	38.56	-19	-5	22	23	22	0.51
MPO	-25	-45	43	25	25	25	1.10
XO	29	21	15	25	25	25	1.81

ABTS•+, 2,2′-azino-bis(3-ethylbenzothiazoline-6-sulfonic acid) diammonium salt radical cation; Keap1, Kelch-like ECH-associated protein 1; MPO, myeloperoxidase; XO, xanthine oxidase.

Table S2: Binding affinities of 29 corn silk peptides docked onto Keap1, MPO and XO.

SPE Fractions	Peptides[a]	Binding Affinity (kcal/mol)[b]		
		**Keap1**	**MPO**	**XO**
0 mM KCl	KRYFKR	-7.8	**-5.5**	-
	PRVRVAGR	-7.9	-4.8	-2.7
	PVVWAAKR	-7.8	**-6.2**	-1.0
	QVASGPLQR	**-8.1**	-5.3	-2.0
	MAPRTPRK	-7.6	-4.6	-
	NKVVKLMR	-7.0	-3.5	-
	KVPLAVFSR	**-8.4**	-5.3	-
	LKKGSPLKR	-7.3	-3.2	-
	FQLKPVFR	-7.8	-4.0	-
	THAVKGVVHK	-7.5	-2.7	-
	YTWKFKGR	**-8.4**	-4.6	-
	ARVPQQSYR	-7.5	-3.8	-
	VHFNKGKKR	-7.0	-3.6	-
	TAPLSSKALKR	-7.0	0.6	-
	FSCPLVMKGPNGLR	-6.3	-	-
20 mM KCl	RHGSGR	-7.9	**-6.2**	-4.8
	NMVPGR	**-8.1**	**-6.6**	-3.7
	FMFFVYK	**-8.2**	-4.9	-
	MCFHHHFHK	-7.5	-1.3	-
200 mM KCl	DFPGAK	**-8.9**	**-6.9**	**-5.2**
	NDGPSR	**-8.0**	**-6.3**	**-5.2**
	AGFPLGK	**-8.4**	**-6.7**	-4.9
	AMQQDK	-7.6	**-5.7**	-3.1
	NLEGYR	**-8.7**	**-6.5**	-3.4
	YETLNR	**-8.5**	**-6.5**	-5.0
	MPPKSTR	-7.8	**-5.7**	-4.0
	TAGASLVAR	**-8.2**	**-5.5**	-2.9
	SSPATGGSLR	**-8.1**	-4.9	-1.5
	NANSLAGPQR	**-8.2**	-3.6	-

[a] Peptides are arranged in order of increasing molecular mass, as in Table 1. [b]Binding affinities in **bold** are the same as or more negative than those computed for DEQIPSHPPR (-8.0 kcal/mol), DTETGVPT (-5.5 kcal/mol) and ACECD (-5.2 kcal/mol), the reference peptides docked on Keap1, MPO and XO, respectively. - indicates positive values, which are not shown. Values presented are mean of four replicates.
